# Using Caprylic Acid for the Prevention and Treatment of *Helicobacter pylori* Infection and Gastric Cancer: A Review

**DOI:** 10.3390/metabo15090629

**Published:** 2025-09-22

**Authors:** Alexandra Balderrama-Gómez, Victor Manuel Muñoz-Pérez, Mario I. Ortiz, Raquel Cariño-Cortés, Javier Castro-Rosas, Abigail Betanzos, Eduardo Fernández-Martínez, Israel Castillo-Juárez

**Affiliations:** 1 Área Académica de Medicina, Instituto de Ciencias de la Salud, Universidad Autónoma del Estado de Hidalgo, Eliseo Ramírez Ulloa No. 400, Doctores, Pachuca 42090, Hidalgo, Mexico; ba197804@uaeh.edu.mx (A.B.-G.);; 2 Área Académica de Química, Instituto de Ciencias Básicas e Ingeniería, Ciudad del Conocimiento, Universidad Autónoma del Estado de Hidalgo (UAEH), Carretera, Pachuca-Tulancingo Km. 4.5, Mineral de la Reforma 42183, Hidalgo, Mexico; 3 Departamento de Infectómica y Patogénesis Molecular, Centro de Investigación y de Estudios Avanzados del Instituto Politécnico Nacional, Ciudad de México 07360, CDMX, Mexico

**Keywords:** medium-chain fatty acids, caprylic acid, goat milk, *Helicobacter pylori*, gastric cancer

## Abstract

The present study investigates the bactericidal and anticancer potential of caprylic acid (CA) against *Helicobacter pylori* infection, a major global risk factor for gastric cancer. Several chronic inflammatory processes, bacterial virulence factors, and carcinogenic mechanisms—capable of inducing DNA damage in gastric epithelial cells, promoting genomic instability, and contributing to the development of gastritis or peptic ulcer disease in susceptible individuals—remain incompletely understood. CA, a medium-chain fatty acid naturally found in plant and animal sources such as coconut oil and goat’s milk, possesses notable biological properties that may confer gastroprotective effects against gastric cancer induced by *H. pylori*. Despite advances in medical management, no universally effective strategy currently exists for the treatment or prevention of *H. pylori*–associated gastric cancer. Conventional therapies, including surgery, radiotherapy, and chemotherapy, often entail long-term complications that may affect patients’ nutritional status. In brief, further elucidation of the mechanisms underlying medium-chain fatty acid metabolism, particularly that of CA in gastric cancer cells, may yield valuable insights for the development of innovative therapeutic approaches. Consequently, the integration of CA into therapeutic dietary regimens and the formulation of nutraceuticals targeting *H. pylori* infection and related gastric pathologies warrant consideration. Therefore, CA could be considered a potential adjuvant in the preventive treatment of *H. pylori*–induced gastritis and its associated complications. However, further in vitro and in vivo studies are needed to confirm its beneficial use for this pathology.

## 1. Introduction

Gastric cancer (GC) represents the fifth most frequently diagnosed malignancy and the third leading cause of cancer-related mortality worldwide. According to the International Agency for Research on Cancer (IARC), more than 1.1 million new cases and approximately 770,000 deaths were recorded in 2020, with a higher incidence observed in males compared to females, although notable regional variations exist. However, projections by the IARC estimate a global burden of approximately 1.8 million new cases and over 1.3 million deaths annually by 2040 [1,2].

*Helicobacter pylori* infection accounts for nearly 90% of distal gastric cancer cases globally [3]. Multiple risk factors contribute to gastric carcinogenesis, including *H. pylori* infection, atrophic gastritis, and pernicious anemia. Additional factors have also been implicated, such as autoimmune gastritis, Epstein–Barr virus infection, and Ménétrier’s disease. Lifestyle-related determinants—including dietary habits, smoking, alcohol consumption, obesity, and socioeconomic status—further modulate the risk of GC development. Accordingly, several prevention and control strategies have been developed, focusing on the eradication of *H. pylori* infection and the promotion of healthy lifestyles. These include reducing tobacco use, obesity, salt and alcohol intake, and increasing dietary vegetable consumption, which together constitute critical components of gastric cancer prevention programs [3,4,5,6].

The pathogenesis of GC is frequently described through Correa’s cascade, a multistep sequence of gastric mucosal changes progressing from chronic non-atrophic or active gastritis to precancerous lesions, and ultimately to adenocarcinoma. This model highlights three phases of malignant transformation: Phase I, chronic active gastritis; Phase II, gastric precancerous lesions; and Phase III, development of gastric carcinoma. Among the etiological factors involved, *H. pylori* plays a central role [5].

This bacterium colonizes the gastric mucosa and directly interacts with epithelial cells, modulating and adapting its microenvironment through the secretion of enzymes and virulence factors [6]. Urease, for example, hydrolyzes urea to neutralize gastric acid, thereby enabling bacterial persistence under acidic conditions. This, in turn, facilitates the expression of virulence factors that damage gastric epithelial cells by disrupting lipid membranes, inducing vacuole formation, and interacting with outer membrane proteins, ultimately leading to mutations during cell replication [6,7].

Key virulence determinants include the vacuolating cytotoxin gene A (VacA), cytotoxin-associated gene A (CagA), urease gene A (UreA), and the cag pathogenicity island (cagPAI). These elements contribute to enhanced urease activity, bacterial adhesion, drug resistance, inflammation, and exacerbation of gastric epithelial damage [7,8]. Chronic *H. pylori* infection therefore represents a major etiological factor in gastric carcinogenesis, with disease progression often occurring after years or decades of colonization [9].

In recognition of its oncogenic potential, the IARC classified *H. pylori* as a Group 1 carcinogen in 1994, a designation reconfirmed in 2009. Its role in gastric carcinogenesis encompasses a pathological continuum beginning with chronic gastritis, advancing to atrophic gastritis and intestinal metaplasia, and potentially culminating in dysplasia and adenocarcinoma [7,10,11,12].

According to current guidelines, eradication therapy should be prioritized for individuals at elevated risk, including those with severe acute gastritis, corpus-predominant gastritis, or a diagnosis of gastric neoplasia. Although *Helicobacter pylori* is theoretically susceptible to a wide range of antibiotics, it has demonstrated a capacity for developing antimicrobial resistance. For instance, clarithromycin–metronidazole–based regimens have generated clinical controversy, as their approval is often limited to specific regulatory jurisdictions and their efficacy is undermined by rising bacterial resistance [4,13].

In general, *H. pylori* eradication therapies achieve satisfactory success rates; however, their impact on epithelial pathology is influenced by the stage of disease progression at the time of treatment initiation. Moreover, treatment efficacy is strongly dependent on patient adherence [4,14]. Importantly, the effectiveness of eradication as a preventive strategy for gastric cancer relies on the early recognition of symptoms, such as epigastric pain, early satiety, postprandial fullness, nausea, vomiting, and, in cases of bleeding, melena, hematemesis, and anemia [15,16]. Furthermore, *H. pylori* infection has been associated with gut microbiota dysbiosis and increasing antibiotic resistance—factors that, if addressed, may improve therapeutic outcomes [15,17,18,19].

For patients with advanced gastric cancer, surgery remains the primary therapeutic intervention, generally followed by chemotherapy and radiotherapy [4,20,21]. Currently, one of the most widely recommended first-line therapies for *H. pylori* eradication consists of a proton pump inhibitor (PPI), such as lansoprazole, in combination with at least two antibiotics, typically clarithromycin and amoxicillin or metronidazole. However, this regimen is frequently associated with adverse effects, including gastrointestinal disturbances, abdominal pain, diarrhea, constipation, dysgeusia, and headache, with or without vomiting [14,22]. In addition, PPIs used in isolation have been reported to produce nonspecific symptoms, such as headache, dermatitis, dizziness, nausea, abdominal discomfort, intestinal bloating, constipation, and diarrhea [23,24]. Consequently, the search for safer and more effective alternatives to eradicate *H. pylori* infection is of critical importance.

In this context, natural products have emerged as promising anticancer agents, particularly in infection-associated malignancies, due to their relative safety, structural diversity, and pleiotropic pharmacological mechanisms—many of which remain underexplored [8,15,25,26]. Current research and development strategies for alternative therapies against *H. pylori*–associated inflammation have focused on plant-derived bioactive compounds and probiotics, both of which exhibit the potential to inhibit bacterial growth and modulate host inflammatory responses [27]. Plant-based compounds may therefore serve not only as preventive agents against *H. pylori*–induced gastrointestinal disorders, but also as adjuncts in functional foods such as coconut oil, where they may help regulate dietary and environmental risk factors while reducing reliance on antibiotic therapies. Consequently, the identification and characterization of novel bioactive compounds for the prevention and management of *H. pylori* infection, with the goal of reducing gastric cancer progression, represents a growing area of scientific interest [28,29].

Indeed, there is increasing demand for novel therapeutic alternatives for *H. pylori* infection and gastric cancer. Natural products—including fruits, vegetables, spices, and herbal remedies—have long been central to traditional medical systems, such as Traditional Chinese Medicine, where they are valued for their efficacy and relatively low incidence of adverse effects [30]. In many developing countries, natural products also constitute an integral component of primary health care [8,31]. Numerous natural compounds with diverse biological activities have already been identified, and many have served as the foundation for the development of modern pharmacological agents [32].

Therefore, this review focuses on the antimicrobial and anticancer properties of caprylic acid (CA), with particular attention to its underlying mechanisms of action. The dietary incorporation of CA offers potential as a preventive and complementary strategy, either as part of therapeutic diets or as a component of nutraceutical development. Finally, this work proposes that regular AC supplementation through diet and nutrition could help prevent *H. pylori* infection while protecting gastric epithelial cells from inflammation and damage, thereby reducing the risk of gastritis and, ultimately, gastric cancer [33].

### 1.1. Medium Chain Fatty Acids: Caprylic Acid

**Figure 1:**
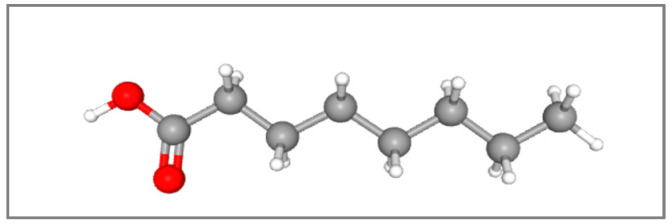
Three-dimensional structure of caprylic acid and its structural formula, H_3_C–(CH_2_)_6_–COOH. 3D model of CA shows a straight-chain molecule with 8 carbon atoms (gray spheres), a methyl group (CH_3_) at one end, a series of six methylene groups (CH_2_) in the middle, and a carboxyl group COOH (red spheres). Adapted from PubChem Compound Summary, CID 379 (National Center for Biotechnology Information, 2025) [43].

Medium-chain fatty acids (MCFAs) are a group of saturated fatty acids containing 6 to 12 carbon atoms. They are characterized by their liquid state at room temperature, relatively low molecular weight, and lower caloric value (8.4 kcal/g) compared with long-chain triglycerides, which provide approximately 9.2 kcal/g [34]. MCFAs are predominantly derived from natural plant and animal sources [35]. They account for approximately 15% of the fatty acid composition of virgin coconut oil, 7.9% of palm kernel oil, and 6.9% of milk. Of particular interest, MCFAs have been widely studied for their diverse biological activities, including antimicrobial properties—effective against pathogenic bacteria and fungi—and anticancer effects in human cells [34,36]. In biological systems, MCFAs are typically present in the form of medium-chain triglycerides (MCTs). Following ingestion, they are absorbed by enterocytes in the small intestine and transported directly into the portal circulation as free fatty acids. In the liver, they undergo rapid mitochondrial uptake and oxidation, yielding Co-A derivatives and ketone bodies [37]. A notable example is caprylic acid (CA), also referred to as octanoic acid. When present as its salt, it is commonly termed caprylate or octanoate. Chemically, CA (C_8_H_16_O_2_) is a saturated fatty acid consisting of an eight-carbon chain (Figure 1) [38]. It is an oily, clear, and colorless liquid with a rancid odor, minimal solubility in water, and a weak acidic character (pKa = 4.89).

CA is naturally found in several plant and animal sources. Among plant oils, cuphea oil contains the highest proportion (≈73%), followed by coconut oil (6–10%) and palm kernel oil (2–5%). Its concentration in animal-derived milks varies considerably, ranging from 0.5% in human breast milk to 1–2% in cow’s milk, 3% in goat’s milk, 5–6% in rat milk, and 15–18% in rabbit milk [39,40].

Currently, caprylic acid is recognized not only as a potential antibacterial agent, but also as an important human metabolite and a metabolite of *Escherichia coli* [40,41,42].

### 1.2. Antimicrobial Properties of Caprylic Acid

The antimicrobial potential of caprylic acid (CA) was first reported in the 1940s and 1950s, when in vitro experiments demonstrated its inhibitory effects on *Candida albicans* [44,45]. In these early studies, complexes of linear saturated fatty acids with resins were evaluated for antimicrobial applications, and complete inhibition of *C. albicans* growth was observed with CA-resin complexes. Subsequent research confirmed this antifungal activity: CA derived from virgin coconut oil exhibited a minimum inhibitory concentration (MIC) of 40 μg/mL against *C. albicans*, highlighting its potential as an antifungal agent [45].

Comparative studies of saturated fatty acids—including CA, lauric acid, myristic acid, palmitic acid, and stearic acid—demonstrated that CA effectively inhibited the growth of *Staphylococcus aureus*, *Escherichia coli*, and *C. albicans* [46]. In porcine jejunal epithelial cells (IPEC-J2) infected with *E. coli*, CA not only exhibited antimicrobial activity in a concentration-dependent manner, but also upregulated endogenous host defense mechanisms, strengthening the intestinal epithelial barrier. These findings suggest that CA may represent a novel approach to preventing bacterial infections and associated gastrointestinal disorders in humans [46].

CA has also shown efficacy against *Listeria monocytogenes*. When combined with potassium sorbate, CA exerted synergistic bactericidal effects on both planktonic and biofilm forms of *L. monocytogenes*, with MIC values ranging from 4 to 128 mg/mL [47]. Similarly, in vitro studies revealed that CA, in combination with organic acids, inhibited foodborne pathogens such as *Salmonella enteritidis*, *L. monocytogenes*, *E. coli* O157:H7, and *S. aureus*, as well as multiple *L. monocytogenes* and *Salmonella* strains isolated from meat-processing environments. Notably, CA exhibited stronger activity at low pH, particularly when combined with acetic and citric acids, and was more effective against Gram-positive than Gram-negative bacteria [48].

Additionally, the antimicrobial role of CA has also been explored against multidrug-resistant (MDR) pathogens. In studies with *Klebsiella pneumoniae*, CA inhibited biofilm formation and, when combined with antibiotics, reduced resistance development, suggesting potential applications as an adjuvant therapy or as a coating for medical devices aimed at preventing *K. pneumoniae* infections [49]. Similarly, the activity of CA against *Helicobacter pylori* has yielded mixed findings. One study reported no bactericidal effect at 1–5 mM concentrations under neutral pH (pH 7) [50], while another demonstrated that antibacterial efficacy is strongly pH-dependent. Specifically, CA exhibited maximal bactericidal effects at 1 mM within pH 2.5–3.0, at 10 mM within pH 4.0–4.5, and at 20 mM at pH 5.0. These discrepancies underscore the influence of experimental conditions, particularly pH, on the antimicrobial activity of CA [51].

**Figure 2:**
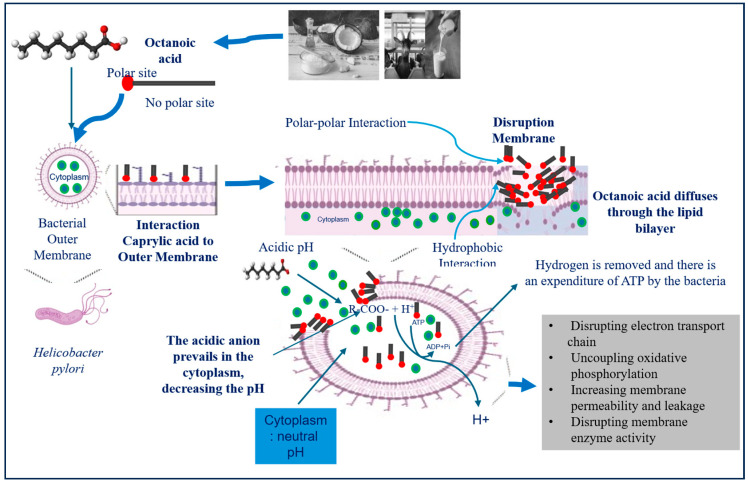
Proposed antibacterial mechanism of caprylic acid (CA). As an amphiphilic molecule, CA interacts with the bacterial outer membrane, increasing membrane permeability. This disruption facilitates CA entry into the cell, leading to metabolic imbalance and ultimately compromising bacterial viability.

The amphiphilic structure of CA likely explains its bactericidal properties [52,53]. With a non-polar eight-carbon aliphatic chain and a polar carboxyl group, CA can penetrate bacterial membranes through passive diffusion. Its hydrophobic tail associates with the lipid bilayer’s non-polar core, while the carboxyl group interacts with polar head groups of phospholipids, leading to membrane disruption (Figure 2). This structural perturbation disrupts ion gradients, impairs oxidative phosphorylation, hinders nutrient transport, and inhibits membrane-bound enzymatic activity, ultimately compromising bacterial viability [3,38,53].

An important consideration is the selective activity of CA against pathogens while preserving beneficial gut microbiota. Fatty acids—including short-chain (SCFAs), medium-chain (MCFAs), and long-chain fatty acids (LCFAs)—play a role in shaping microbial ecology by preventing pathogen colonization and modulating microbiota composition. Alterations in gut microbiota can compromise intestinal barrier function and trigger systemic inflammation [54]. The human gut microbiota is predominantly anaerobic, comprising mainly the phyla *Bacteroidetes* (9–42%), *Firmicutes* (30–52%), and *Actinobacteria* (1–13%), with smaller proportions of *Lactobacillales* (≈2%) and *Enterobacteriaceae* (≈1%) [54].

SCFAs such as acetate, propionate, and butyrate are fermentation products of *Bacteroidetes*, *Negativicutes*, and *Firmicutes*, and are associated with beneficial effects including improved gut health, obesity prevention, and metabolic regulation. Similarly, MCFAs—such as caprylic (C8:0), capric (C10:0), and lauric (C12:0) acids—derived from coconut oil, palm oil, and human milk, are rapidly absorbed and transported to the liver, where they regulate energy metabolism, enhance insulin sensitivity, and promote satiety. They may also elongate to long-chain fatty acids and reassemble into triglycerides. Importantly, MCFAs have been shown to increase *Bacteroidetes* populations in the gut microbiota and stimulate anti-inflammatory effects in obese mice. Specifically, CA promotes the production of lactate and acetate by beneficial gut bacteria, thereby enhancing host protection against enteropathogens [54,55].

### 1.3. H. pylori in Gastric Cancer

The anticancer properties of natural products remain an area of great interest of the international scientific community, particularly when such compounds demonstrate both health-promoting effects and applicability in cancer therapy. Their relevance lies in the ability to target multiple molecular pathways simultaneously, including pathophysiological and pharmacological processes implicated in neoplastic diseases of infectious origin, such as *Helicobacter pylori*–associated gastric cancer [56].

Several studies have highlighted the beneficial effects of dietary fatty acids—including short-chain, medium-chain, and long-chain fatty acids—on tumor biology. These compounds have been reported to modulate inflammation, metastasis, and tumor progression in in vitro gastric cancer cell lines, as well as in in vivo and ex vivo models of *H. pylori* infection. In particular, medium-chain fatty acids (MCFAs) have demonstrated the capacity to suppress tumor growth in xenograft models of human gastric adenocarcinoma cells and to prolong animal survival [49,57,58,59].

**Figure 3:**
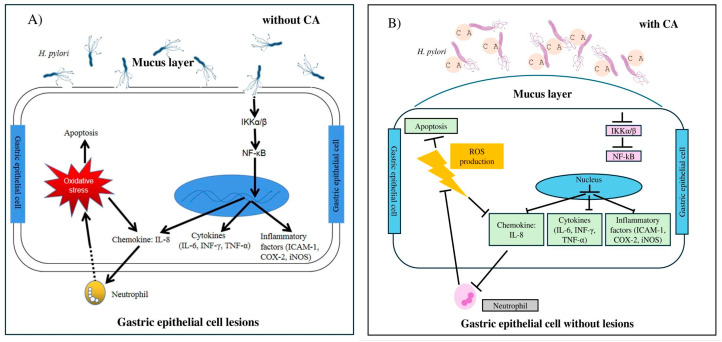
Proposed immune response during *H. pylori* infection via activation of the NF-κB signaling pathway. (**A**) Colonization of gastric epithelial cells by *H. pylori* initiates a cascade of molecular events. Activation of IκB kinases (IKKα/β) leads to the release and nuclear translocation of NF-κB, inducing the expression of pro-inflammatory mediators such as ICAM-1, COX-2, and iNOS, as well as cytokines, including IL-6, IFN-γ, and TNF-α, and chemokines such as IL-8. This process promotes immune cell recruitment, oxidative stress, and ultimately apoptosis. (**B**) CA inhibits *H. pylori* adhesion to epithelial cells, thereby attenuating the inflammatory response, preventing immune cell infiltration, and reducing apoptosis. Abbreviations: NF-κB, Nuclear factor-kappa B; IKKα/β, IκB kinase α/β; IFN-γ, Interferon-γ; TNF-α, Tumor necrosis factor-α; IL, Interleukin; ICAM-1, Intracellular adhesion molecule-1; COX-2, Cyclooxygenase-2; iNOS, Inducible nitric oxide synthase; ROS, Reactive oxygen species. (Adapted from [56], licensed under the Creative Commons Attribution License).

*H. pylori* is a well-established pathogen in chronic gastritis, capable of inducing long-term inflammatory responses through the release of pro-inflammatory mediators such as tumor necrosis factor-alpha (TNF-α) and interleukin-1 (IL-1) (Figure 3). Sustained production of these mediators contributes to cellular injury, genetic mutations, epigenetic alterations, and dysregulation of local cellular function, ultimately increasing the risk of neoplastic transformation [60]. Moreover, *H. pylori* is implicated in the initiation phase of gastric carcinogenesis, with established associations to gastric and duodenal ulcers, as well as the development of mucosa-associated lymphoid tissue (MALT) lymphoma [61].

The bacterium’s ability to colonize long-term, modulate host immune responses, and induce gastric pathology underscores its role as a unique oncogenic pathogen. Epidemiological observations suggest that only a subset of infected individuals progress to gastric cancer, likely influenced by host immune responses and the intensity of localized inflammation within the gastric epithelium [61,62].

In this regard, as a Gram-negative bacterium, *H. pylori* secretes outer-membrane components such as lipopolysaccharide (LPS), a major virulence factor and microbial mediator implicated in the pathogenesis of sepsis and septic shock. LPS activates mononuclear immune cells through Toll-like receptor 4 (TLR4), triggering the production of pro-inflammatory mediators such as TNF-α and IL-1. This response promotes the formation of microthrombi and chronic inflammation, processes linked to a wide spectrum of metabolic and inflammatory diseases, including gastric cancer, atherosclerosis, type II diabetes, obesity, and neuroimmune disorders [63].

Notably, the LPS of *H. pylori* differs from that of other Gram-negative bacteria, such as *E. coli*, in its immune-modulatory properties. While it induces the release of pro-inflammatory cytokines (TNF-α, IL-1β, IL-6, and IL-8), it also selectively activates anti-inflammatory pathways. Specifically, *H. pylori* LPS upregulates IL-8 and IL-12 while simultaneously inactivating other cytokines, leading to the suppression of T-cell–mediated immune surveillance. This dual mechanism allows for immune evasion and facilitates the initiation and progression of gastric cancer—an established hallmark of tumor cell escape strategies [64].

Interestingly, dietary components such as probiotics, vegetables, and certain oils have shown protective effects against *H. pylori* infection and are increasingly regarded as natural therapeutic alternatives. Among these, goat milk has long been recognized for its medicinal value, primarily due to its content of three medium-chain fatty acids: capric, caprylic, and caproic acids. The anticancer potential of these fatty acids has been demonstrated in several cell lines, including HCT-116 (human colorectal carcinoma), A-431 (human squamous cell carcinoma), CCD-33Co (normal colon fibroblast), and MDA-MB-231 (human breast adenocarcinoma) [56,65].

Recently, several in vitro and in vivo studies have reported novel findings regarding the relationship between *H. pylori* infection and its potential clinical implications in other neoplastic malignancies. These include esophageal cancer associated with Barrett’s esophageal adenocarcinoma, colorectal cancer examined in Foxp3 IL-17A T cells and in C57BL/6 mice infected with *H. pylori*, liver cancer in infected patients, as well as pancreatic and gallbladder cancers linked to this pathogen. Conversely, it has been suggested that *H. pylori* infection may contribute to gastric mucosal atrophy and reduce gastric acid secretion, thereby decreasing the incidence of Barrett’s esophagus (BE) caused by gastroesophageal reflux disease and subsequently reducing the risk of esophageal adenocarcinoma. The association of *H. pylori* with diverse gastrointestinal cancer could inform the development of new preventive and pharmacological strategies for early detection and diagnosis, highlighting the need for updated clinical guidelines [66].

Dietary sources such as goat milk and coconut oil provide medium-chain fatty acids (MCFAs), which are considered nutraceuticals due to their cancer-preventive properties [67]. Unlike long-chain fatty acids, MCFAs are transported into mitochondria independently of carnitine, thereby enhancing cellular energy metabolism. Specifically, CA has demonstrated anticancer activity by inhibiting cell migration, adhesion, proliferation, and invasion in various cancer models, including skin, bladder, colon, and breast cancer cells. Its therapeutic potential in *H. pylori*–associated gastric cancer is partly attributed to its modulation of the pro-inflammatory cytokine TNF-α, a key regulator of inflammation and T-cell responses in both acute and chronic immune-mediated conditions [63,67,68,69,70].

The pro-inflammatory cytokine interleukin-6 (IL-6) is involved in stimulating the production of acute-phase proteins during inflammation and promotes the generation of neutrophils in the bone marrow. Notably, CA appears not to exacerbate inflammation. In endothelial cells and monocytes stimulated with TNF-α to mimic sepsis, caprylate enhanced mitochondrial respiration without increasing IL-6 secretion, suggesting that it may influence energy metabolism in cancer and normal tissues without promoting pro-inflammatory signaling [71]. This metabolic effect supports the hypothesis that CA can reduce available energy sources in tumor cells while sparing healthy tissues [68,70,72].

**Figure 4:**
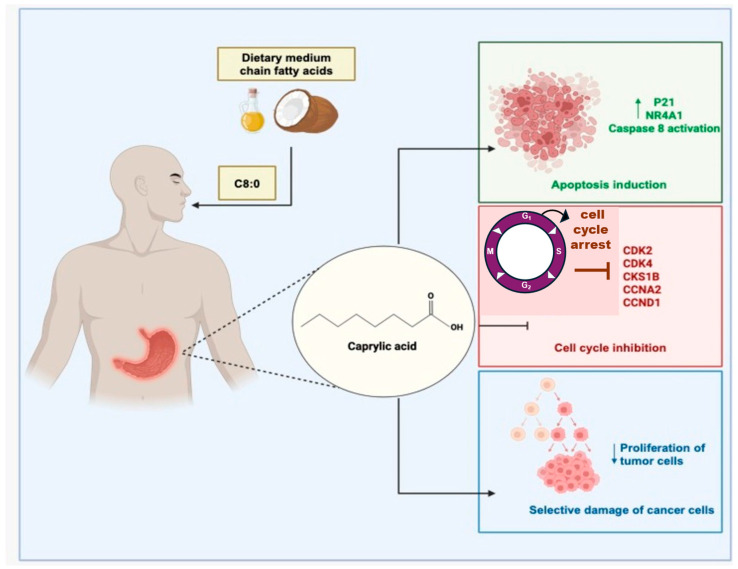
Proposed mechanism of action of caprylic acid in gastric cancer. CA exerts multiple anticancer effects through distinct molecular mechanisms. (**Green box**) CA induces apoptosis in gastric cancer cells via upregulation of *P21* and *NR4A1* expression, as well as caspase-8 activation. (**Red box**) CA inhibits cell cycle progression by targeting cyclin-dependent kinases (CDK-2, CDK-4) and their associated regulators (CKS1B, CCNA2, CCND1), thereby blocking uncontrolled proliferation. (**Blue box**) CA decreases tumor cell proliferation.

Interesting, a wide range of phytochemicals also exhibit chemopreventive properties through activation of apoptotic pathways in premalignant and malignant cells. These compounds interfere with multiple steps of tumor progression, including growth, invasion, and metastasis [73]. However, because cancer progression is regulated by complex, multifactorial mechanisms, effective treatment often requires targeting multiple molecular pathways simultaneously. Plant-derived compounds and their derivatives are being actively investigated as anticancer agents, with several already in clinical trials. One of the most critical hallmarks of cancer—resistance to apoptosis—can be overcome by natural compounds that modulate intrinsic and extrinsic apoptotic pathways, induce ferroptosis, regulate the cell cycle, and inhibit proliferation [74,75]. The potential molecular mechanism underlying the anticancer effects of caprylic acid (CA) has been linked to the downregulation of genes involved in cell cycle regulation and apoptosis in tumor cells. Genes encoding proteins such as cyclin-dependent kinase 2 (CDK2), cyclin-dependent kinase 4 (CDK4), CDC28 protein kinase 1B (CKS1B), cyclin A2 (CCNA2), and cyclin D1 (CCND1) play key roles in promoting cell division and cancer progression. However, CA appears to suppress the expression of several of these genes, including CKS1B, CCNA2, and CCND1, while simultaneously promoting the upregulation of tumor-suppressor genes, such as peroxiredoxin 1 (PRDX1) and cyclin-dependent kinase inhibitor 1 (CDKN1A, also known as P21). P21 is known to inhibit the activity of various cyclin-CDK complexes -such as cyclin A–CDK2, cyclin D1–CDK4, and cyclin E1–CDK2- resulting in cell cycle arrest at the G1/S transition checkpoint. This mechanism has been linked to CA’s reported efficacy in glioblastoma models, where it inhibited tumor growth and displayed neuroprotective and mitochondria-protective effects in models of neurodegenerative disease (Figure 4) [72,75,76].

Additionally, CA derived from goat milk has been shown to activate the extrinsic apoptotic pathway via caspase-8 activation in both premalignant and malignant colon cancer cells [77]. Together, these findings emphasize the importance of developing and clinically validating novel natural-origin anticancer agents, which may offer therapeutic benefits while minimizing cytotoxicity and side effects.

Recently, Cachexia in advanced cancer has recently attracted growing attention, particularly due to its frequent association with cardiac complications, including myocardial dysfunction. In this context, natural products such as fatty acids have been studied in animal models of cancer-associated cachexia to elucidate the role of inflammatory cytokines—including IL-1, IL-6, IL-8, TNF-α, and the nuclear protein high-mobility group box-1 (HMGB1)—in promoting catabolism and systemic inflammation [78]. Overexpression of HMGB1 in gastric and colon cancer promotes invasion and metastasis by suppressing antitumor immune responses. Elevated circulating HMGB1 and TNF-α levels have been reported in cachectic cancer patients, correlating with skeletal muscle sarcopenia. This process is mediated through binding to receptors for advanced glycation end products (RAGEs) and TLR-4, which trigger autophagy and lead to myocardial atrophy [78].

However, medium-chain fatty acids, particularly caprylic acid (CA), appear to mitigate these deleterious effects by reducing systemic inflammation and protecting myocardial tissue. Experimental studies suggest that CA decreases mitochondrial oxidative stress, lowers reactive oxygen species (ROS) levels, and reduces circulating TNF-α, troponin II, and HMGB1. Collectively, these changes have been linked to reduced HMGB1 expression and attenuation of cachexia, as reported in animal models [79]. Furthermore, CA activates AMP-activated protein kinase (AMPK), a key regulator of cellular energy homeostasis, thereby preserving normal cardiac and skeletal muscle function. Through enhanced mitochondrial metabolism of MCFAs, CA promotes the generation of ketone bodies, which serve as alternative energy substrates for muscle tissue. In addition, CA downregulates TLR-4, NF-κB, TNF-α, and other pro-inflammatory mediators. These properties suggest that CA could have therapeutic potential in other malignancies associated with paraneoplastic syndromes involving metabolic, immunologic, or non-immunologic mechanisms [78,80].

## 2. Methods

### Data Sources

An electronic bibliographic search was carried out from databases and indexing services including Scopus, PubMed, Cochrane Library, Embase, Web of Science and Google Scholar addressing *Helicobacter pylori*, gastric cancer, caprylic acid, medium chain fatty acids (MCFA), action mechanism of CA on *H. pylori*, among others; however, databases were systematically searched to identify relevant studies published up to June 2025.

## 3. Conclusions

*Helicobacter pylori*–induced inflammation in gastric cancer promotes epithelial cell mutations, alterations in gene expression and epigenetic regulation, changes in cell signaling pathways, genomic instability, and apoptosis, ultimately driving the progression from pre-neoplastic to neoplastic lesions. *H. pylori*–related mucosal inflammation is closely linked to gastric carcinogenesis, particularly through the activity of virulence factors such as CagA and VacA, which have recently emerged as molecular markers, enabling the identification of patients with early *H. pylori* infection and associating both factors with gastric cancer progression and development in its earliest stages. This capability allows for the prediction of GC prognosis and the design of targeted therapies [81].

Medium-chain fatty acids (MCFAs), naturally present in the diet, have emerged as important modulators of bacterial infections and metabolic pathways implicated in various cancers. Gastric cancer remains the third leading cause of cancer-related mortality worldwide, underscoring the need for preventive interventions. Evidence suggests that a balanced diet with moderate MCFA intake may reduce cancer risk. Given their unique biological and chemical properties, dietary MCFAs have the potential to influence human health by preventing or mitigating *H. pylori* infection and subsequent gastric carcinogenesis [40,66,79].

One notable advantage of MCFAs is their capacity to regulate inflammatory gene expression in infectious and non-infectious diseases, including through inhibition of PPAR β/δ signaling pathways [80]. Among MCFAs, caprylic acid (CA) has been particularly highlighted for its antimicrobial and anticancer properties. CA is proposed as a potential adjuvant for managing inflammation and infections associated with *H. pylori*. Dietary sources such as coconut oil, palm oil, and milk from select mammals can contribute to CA intake, potentially supporting the prevention and treatment of microbial infections [40,66,79,80].

Mechanistically, MCFAs like CA penetrate bacterial membranes in their protonated (acidic) form, leading to intracellular acidification and activation of fatty acid transport mechanisms. Upon reaching a critical intracellular concentration, CA exerts a bactericidal effect. This process was initially observed in *Saccharomyces cerevisiae*, where intracellular acidification enhanced plasma membrane H^+^-ATPase activity and facilitated CA entry [82]. Similarly, CA disrupts the membrane integrity of Gram-negative bacteria, such as enteric *Campylobacter*, through passive diffusion, causing cytoplasmic acidification and impairing bacterial viability [83]. These antimicrobial effects suggest that CA may interfere with *H. pylori* colonization and adherence to gastric epithelial cells, thereby offering protection against gastritis, ulcers, and gastric cancer.

Dietary supplementation with CA may prevent *H. pylori* adhesion to gastric epithelial cells, thereby reducing the risk of gastritis, ulcers, and GC. Its combined antimicrobial and anti-inflammatory properties make CA a promising nutritional adjuvant in pharmacological strategies for the prevention and management of gastric diseases. Early inclusion of CA-rich foods, such as coconut oil, palm oil, and goat’s milk, in the diet may confer long-term protective effects, not only against gastrointestinal disorders, but also against autoimmune diseases and colitis. This approach could inform the development of early-life nutritional education programs, promoting healthy dietary habits and fostering a culture of nutrition from childhood.

However, although CA is considered a potential adjunct in inhibiting *H. pylori* growth in vitro and in vivo, it appears to have some challenges in implementing this new knowledge to preventive human therapies due to pharmacological limitations such as the lack of findings exploring potential adverse and side effects. Additionally, how can we prove experimentally in humans the ability of CA to disrupt the cell membrane of *H. pylori* and interfere with its ability to colonize the stomach? Demonstrating these effects experimentally in humans is complex, and translating them into measurable clinical benefits will require rigorous investigation. Addressing these gaps could facilitate the development of innovative adjunctive pharmacological interventions for H. pylori-induced gastric cancer, incorporating CA as a preventive and therapeutic agent.

## Data Availability

No new data were created or analyzed in this study.

## Abbreviations

The following abbreviations are used in this manuscript:

A-431	human skin squamous cell carcinoma cells
CA	caprylic acid
CagA	cytotoxin-associated gene A
cagPAI	the cag pathogenicity island
CCD-33Co	normal human colon fibroblast
CCNA2	cyclin A2
CCND1	gene encodes the protein cyclinD1
CDC 28	protein kinase regulatory subunit 1B
CDK	cyclin-dependent kinase
CDK2	cyclin-dependent kinase 2
CDK4	cyclin-dependent kinase 4
CID	compound ID
CKSIB	cyclin-dependent kinase regulatory subunit 1B
CoA	coenzima A
COX-2	cyclooxygenase-2
DNA	deoxyribonucleic acid
GC	gastric cancer
GI	gastrointestinal problems
G1-S	G1 phase to S phase transition
HCT-116	human colorectal carcinoma cells
IARC	the International Agency for Research on Cancer
ICAM-1	Intracellular adhesion molecule-1
IKKα/β	IκB kinase α/β
IL-6	interleukin-6
IL-1	Interleukin-1
IL-8	Interleukin-8
INF-γ	Interferon-γ
iNOS	Inducible nitric oxide synthase
IPEC-J2	porcine jejunum epithelial cells
MALT-Lymphoma	mucosa-associated lymphoid tissue lymphoma
MCFA	medium-chain fatty acids
MDA-MB-231	human mammary gland adenocarcinoma cells
MDR	develop multidrug resistance
MIC	minimum inhibitory concentration
NF-κB	nuclear factor-kappaB
NR4A1	nuclear receptor 4A1
P21	cyclin-dependent kinase inhibitor 1
pH	hydrogen potential
pKa	the negative logarithm of the acid dissociation constant (Ka) of a molecule
PPI	proton pump inhibitor
PS	potassium sorbate
ROS	reactive oxygen species
SS1	Sydney Strain 1
TNF-α	tumor necrosis factor-alpha
VacA	vacuolating cytotoxin
